# Trackformers: in search of transformer-based particle tracking for the high-luminosity LHC era

**DOI:** 10.1140/epjc/s10052-025-14156-3

**Published:** 2025-04-25

**Authors:** Sascha Caron, Nadezhda Dobreva, Antonio Ferrer Sánchez, José D. Martín-Guerrero, Uraz Odyurt, Roberto Ruiz de Austri Bazan, Zef Wolffs, Yue Zhao

**Affiliations:** 1 High-Energy Physics, Radboud University, Nijmegen, The Netherlands; 2 Institute for Computing and Information Sciences, Radboud University, Nijmegen, The Netherlands; 3 Faculty of Engineering Technology, University of Twente, Enschede, The Netherlands; 4 Institute of Physics, University of Amsterdam, Amsterdam, The Netherlands; 5 National Institute for Subatomic Physics (Nikhef), Amsterdam, The Netherlands; 6 SURF, Amsterdam, The Netherlands; 7 Intelligent Data Analysis Laboratory (IDAL), Department of Electronic Engineering, ETSE-UV, University of Valencia, Valencia, Spain; 8 Instituto de Física Corpuscular, University of Valencia, Valencia, Spain; 9 Valencian Graduate School and Research Network of Artificial Intelligence (ValgrAI), Valencia, Spain

## Abstract

High-Energy Physics experiments are facing a multi-fold data increase with every new iteration. This is certainly the case for the upcoming High-Luminosity LHC upgrade. Such increased data processing requirements forces revisions to almost every step of the data processing pipeline. One such step in need of an overhaul is the task of particle track reconstruction, a.k.a., *tracking*. A Machine Learning-assisted solution is expected to provide significant improvements, since the most time-consuming step in tracking is the assignment of hits to particles or track candidates. This is the topic of this paper. We take inspiration from large language models. As such, we consider two approaches: the prediction of the next word in a sentence (next hit point in a track), as well as the one-shot prediction of all hits within an event. In an extensive design effort, we have experimented with three models based on the Transformer architecture and one model based on the U-Net architecture, performing track association predictions for collision event hit points. In our evaluation, we consider a spectrum of simple to complex representations of the problem, eliminating designs with lower metrics early on. We report extensive results, covering both prediction accuracy (score) and computational performance. We have made use of the REDVID simulation framework, as well as reductions applied to the TrackML data set, to compose five data sets from simple to complex, for our experiments. The results highlight distinct advantages among different designs in terms of prediction accuracy and computational performance, demonstrating the efficiency of our methodology. Most importantly, the results show the viability of a one-shot encoder-classifier based Transformer solution as a practical approach for the task of tracking.

## Introduction

Today’s scientific research, especially in experimental physics, always involves computational components. In fields such as experimental High-Energy Physics (HEP), data-intensive analysis is essential. Consequently, data science and Machine Learning (ML) techniques have become an integral part of these analyses. The application of deep learning in HEP offers several opportunities for the incorporation of new network architectures that may significantly improve prediction accuracy, computational efficiency, or both. Our focus is on the use of the *Transformer* ML model architecture [1] and the *U-Net* architecture [2] to overcome the challenge of tracking in HEP.

*Tracking* refers to the reconstruction of the trajectory (track) of subatomic particles present in particle physics experiments. The task of tracking involves reconstructing the trajectory of a particle based on detector data. A sensor may or may not be *hit* by a particle, and sequences of these hits comprise tracks. In HEP, the sensory data can come from detectors such as, ALICE [3], ATLAS [4], CMS [5], and LHCb [6], installed at the Large Hadron Collider (LHC). At the same time, tracking is important for experiments in neutrino physics, astroparticle physics and other fields, e.g., [7–10].

Since the tracking algorithms are time-consuming for most experiments, the reconstruction of events is mainly done offline, i.e., after data acquisition. The reconstruction of the trajectories of charged particles often takes place in two steps. In the first step, the hits of the sensors belonging to this particle are identified, and in the second step, the kinematic properties of the particle trajectory are determined using a mathematical model of the possible trajectories. This paper deals with novel methods for the first of these two steps, i.e., the fast and accurate assignment of hits to particles or track candidates.

Traditional tracking methods, such as Kalman filters [11, 12] are currently fundamental in particle tracking for the LHC. These approaches rely on statistical modelling to predict the future state of a particle based on its past states using an iterative process and assuming Gaussian uncertainties. Interestingly, they can be considered as autoregressive models and therefore have properties similar to large language models that predict the next state based on the previous states. With the increase of the integrated luminosity expected during the High-Luminosity phase of the LHC, the number of tracks and the density of detected hits are correspondingly expected to increase significantly. This makes the discrimination between overlapping tracks more difficult, so that more sophisticated and above all faster algorithms are needed to maintain the accuracy of tracking. Track reconstruction, in its current form, is also one of the most computationally intensive components of event reconstruction, as it exhibits quadratic growth with the number of particles in the detector. There is therefore a real need for faster solutions that can be deployed for the upcoming High-Luminosity upgrade of the LHC.

### Our contribution

This paper focuses on the development of first solutions for the assignment of hits to track/particle candidates using the *Transformer* and the *U-Net* ML model architectures. Note that for our current solutions, we have opted to address the formation of hit clusters matching tracks and do not intend to cover the actual parameterisation of a track function, which would be a separate non-trivial task. The challenge is to achieve high solution accuracy, which corresponds to the accuracy of assigning hits to correct tracks, while achieving better computational efficiency. Here, computational efficiency is measured by the mean execution time per event.

Inspired by *large language models* predicting the next word from a large set of words (a certain vocabulary), we have developed an autoregressive Transformer encoder-decoder model that predicts the next hit from a set of hits (EncDec). We argue however that the inference time of such a model scales with the total number of hits, or worse. Therefore, we have also investigated methods that can assign particle/track labels to all hits within an event, *in a single step*. Such models can be considered as “translation” or “mapping” models, translating sensor hits into track candidates, in one step. Accordingly, we have developed a Transformer encoder-based track classifier that maps or translates hits into track candidates (EncCla). Additionally, we have developed a U-Net based architecture, inspired by models assigning labels in images, in a single step.

As an alternative approach, we have developed a model that translates hits into track parameters using a Transformer encoder-based track regressor (EncReg). This approach is conceptually similar to the Hough Transform [13], a classic method used in image processing to detect shapes like lines and circles by mapping points to a parameter space. Similarly, the EncReg model predicts track parameters directly from the hit data. To assign labels to the hits, our model is followed by a clustering algorithm that clusters hit candidates in the track parameter space. This combined approach allows for effective track reconstruction by first mapping hits to parameters and then grouping them into track candidates.

Our goal is to offer fast, i.e., computationally-efficient and high-throughput, solutions for tracking. Therefore, we strive for models that assign hits to trajectories in a single inference step. We determine the accuracy and speed of these models for the tracking problem and use simulated data of increasing complexity to compare our approaches. Finally, we discuss how our initial approaches can improve their performance through post-processing steps and argue that our approaches are fast and accurate enough to be considered as candidates for future tracking pipelines. Our data sets [14] and code [15]^1^ are publicly available.

### Advances and applications in particle tracking for high-energy physics

Particle tracking is a crucial task in HEP experiments. Over the years, various methods have been developed and refined to enhance the accuracy and efficiency of particle tracking.

#### HEP experiments and tracking

In this context, by HEP experiments, we refer to accelerator experiments in which high-energy subatomic particle collisions occur. The Large Hadron Collider (LHC) is perhaps the most well-known particle accelerator, and provides the highest-energy collisions to date. In the LHC, either protons or ions are made to smash into one another in so-called *events*. In this paper, we focus our attention on proton-proton (*pp*) collision events.

These events in turn release a plethora of subatomic particles for which the behaviour is studied through *tracking* and *calorimetry*. Sophisticated detectors, such as ALICE [3], ATLAS [4], CMS [5], and LHCb [6], allow us to measure the footprint of individual particles as they travel through space. They are each equipped with dedicated tracking detectors designed to measure the trajectories of charged particles. These consist of layers of sensitive material, such as silicon detectors, generating electrical signals—hits—when charged particles pass through them. These hits are not continuous, but discrete recordings and limited by detector density.

#### Simulations for HEP

Any research/design work with the aim of developing algorithmic solutions or improving existing algorithms requires large amounts of (labelled) data. These data sets are to be used for extensive testing and validation of the algorithms’ expected characteristics, such as correctness, data processing capacity and performance, computational efficiency and power consumption. The same applies, or rather is strictly required, when it comes to solutions involving ML models.

As HEP experiments are not of the kind to be performed on demand, simulations are suitable and often necessary alternative. Simulations for HEP can be used to study the effects of physics phenomena through the generation of data sets for analyses and algorithm design efforts. There are numerous simulations available, predominantly focusing on physics-accuracy and detector specificity. Examples relevant to the ATLAS detector are Geant4 [16], FATRAS [17] and ATLFAST [18].

#### Traditional methods and performance

Traditional methods to assign hits to track candidates include the Kalman Filter (KF) and Hough Transforms. The KF assumes Gaussian uncertainties and is considered an optimal solution under certain conditions, although it can be slow. The KF is an algorithm that determines the internal state of a linear dynamic system by recursively processing discrete measurements with random perturbations present in the measurements and the system itself [19]. Both stages of tracking rely on the KF [20], with the track finding phase using a specific version called the combinatorial KF (CKF). This starts with a track seed (a recorded hit) and updates it with the information from subsequent hits. The next hit is selected from a pool of candidates, evaluated, and scored to find the most fitting one. In the track fitting stage, a standard KF algorithm is used, but the overall procedure is similar.

Variations of Kalman filtering have been used at LHC for decades [11, 20, 21] (and similar GPU-based algorithms [22]) due to its robustness and excellent performance. However, its combinatorial nature in the track finding stage and inherent sequential execution make KF unsuitable for the upcoming High-Luminosity stage of the LHC, as it scales poorly with the number of recorded hits. The currently utilised algorithm is tested on simulated data of events with 200 points of origin of tracks (pile-up) [23], which is similar in complexity to the largest data evaluated in this paper. The KF pipeline takes 214.3 HS06 $\times$ seconds for a single event, translating to around 12 s CPU-time.^2^ An optimised algorithm with tighter track selection in the track finding stage achieves a 7x speed-up, requiring 1.8 s of CPU-time per event [23].

#### Machine learning competitions and community engagement

To engage data scientists and machine learning experts, a tracking competition (TrackML) [24] was launched on the Kaggle platform. A training data set was created based on the simulation of a generic High-Luminosity LHC (HL-LHC) experiment tracker, listing the measured 3D hits/points for each event and the list of 3D hits belonging to a real track. The events are top-pair events with a “pile-up” of $\mu =200$ using Poisson statistics for the superposition of Quantum ChromoDynamics (QCD) events, in which, there are concentrations of events in close proximity.

#### Advances in deep learning and GNNs

Recent advances in deep learning have led to the adoption of Deep Neural Networks (DNNs) for particle tracking. These models can learn complex, non-linear relationships from data, potentially improving track reconstruction accuracy. Various architectures, including Convolutional Neural Networks (CNNs) and Recurrent Neural Networks (RNNs), have also been explored for this purpose.

Given that particle interactions can be conveniently represented as graphs, Graph Neural Networks (GNNs) have emerged as a promising approach. These networks operate on graph-structured data, capturing the relational information between hits in a detector. The state-of-the-art in recent years has focused on GNN-based solutions [25–38], or see this reference for a review on GNNs in HEP [39].

In GNN-based methods, edges between vertices (hits) are predicted to determine actual physical trajectories. A graph is generated based on an event, with all hits as nodes connected by edges based on some constraint.^3^ A GNN is trained to assign weights to edges or prune unlikely ones, ensuring a fitting connectivity between vertices to represent particle trajectories [28, 34, 40].

A recent successful approach [41, 42] involves constructing a graph by connecting hits from different detector layers that satisfy certain geometric constraints. A fully connected neural network estimates the weights of all edges, pruning those below a threshold. Next, object condensation clusters hits of the same track in a learned space, regressing the properties of the reconstructed objects [43]. Clustering is done using Density-Based Spatial Clustering of Applications with Noise (DBSCAN). Performance is assessed on the inner detector hits from the TrackML data. Lieret et al. [42] defined multiple custom metrics to assess physics performance. No time performance is reported, but the authors suggest that using Transformer models would significantly reduce inference time. An interesting benchmark for GNNs is [34], which reports an inference time of 2.2 s wall-clock time (including data transfer to GPUs) on the full TrackML events, using an NVIDIA A100 GPU. This approach involved significant pre and post-processing of the data and reports a TrackML score of about 0.87. In our approach, we consider an iteration of the TrackML score, i.e., FitAccuracy, as a metric for scoring the success rates of our solutions. FitAccuracy score and its relation with TrackML score is described in detail in Sect. 4.

Finally, we would like to point out that transformers have already been used in many applications in HEP and have proven their versatility and efficiency [44–49]. Though there are more visionary efforts towards the use of Transformers for foundational models, or to further generalise their application in collider physics, these efforts are not as mature yet [50].

#### Software frameworks and other ML-assisted solutions

Other approaches to improve tracking include software frameworks like “A Common Tracking Software (ACTS)” [51], tested on the Open Data Detector (ODD). ACTS is an experiment-independent toolkit for particle track reconstruction in HEP.

ML-assisted solutions have led to specific improvements, such as ambiguity resolution at the end of the tracking chain, determining which track candidates to keep or discard [52]. This insight generation can be considered a side benefit of researching ML-assisted solutions. Incorporating data-driven, iterative improvements to traditional algorithms or partial inclusion of ML models is a conservative approach. We seek a solution predominantly relying on ML models as its core building block, operating as a single-pass algorithm. As in many computer science topics, there is also the question of monolithic versus hybrid/modular deployment of ML models. A promising detector-specific hybrid solution focuses on finding Primary Vertices (PVs) [53, 54]. The approach of replacing parts of traditional tracking solutions will eventually lead to hybrid/modular solutions.

#### Data reduction and standardisation

The most notable trend is the de facto use of TrackML [55] data for experimentation. However, ML model training is computationally expensive and requires large amounts of hardware resources, such as GPU memory. Therefore, authors often reduce the data. The most common reduction is to only consider data associated with the inner detector, also known as the pixel detector [27, 28, 30, 32, 33, 36, 37]. A few authors apply further reductions, such as noise hit reduction [27, 33] or filtering for limited $P_T$ values [32]. Some examples consider the full detector but apply lower pile-up alongside noise hit reduction [35].

The application of reduction to a de facto standard data set (TrackML) and the lack of consensus for a common data reduction protocol, render the data used by different authors non-standard. This makes it challenging to perform direct comparisons between different results. Our data reduction protocol and the considered performance metrics are elaborated in Sects. 2 and 4, respectively.

## Data sets

In this section, we describe the data sets used to train and evaluate our particle tracking methods. These data sets cover a spectrum of scale and track representation complexity, ranging from simple linear tracks to more complex helical and closer to real-world tracks. By increasing the problem complexity in two dimensions (scale and track representation) we can thoroughly assess the performance and robustness of the tracking algorithms. As such, we consider five data sets, covering a spectrum of scale and track representation complexity from low to high. Data set titles are,

- 10–50 (variable count) linear tracks per event, generated with REDVID,
- 10–50 (variable count) helical tracks per event, generated with REDVID,
- 50–100 (variable count) helical tracks per event, generated with REDVID,
- 10–50 (variable count) tracks per event, extracted from the TrackML data set,
- 200–500 (variable count) tracks per event, extracted from the TrackML data set.

### REDVID data set

The first three data sets are the result of simulations using REDVID simulation framework [56]. REDVID simulations are fully configurable with an extensive set of options. We have considered events with random track counts. Simulations with minimum and maximum track count boundaries as [10, 50] and [50, 100] have been executed. Track function complexity varies between linear and expanding helical, with the latter representing a simple emulation of charged particles in a magnetic field. Note that the 3D geometric space and elements contained within are defined in cylindrical coordinate system, with *r*, $\theta$ and *z* coordinates as radius, angle with the X-axis and location on the Z-axis, respectively. The above REDVID data sets contain 100,000 events each, with noise (smearing) applied to calculated hit coordinates. Data set headers include 15 fields, covering

- event, sub-detector, track and hit ids,
- track function parameters, whether linear or helical, same number of parameters are present, with different interpretations per track type,
- hit coordinates, and
- a few type descriptors for sub-detector and track types.

For more detailed descriptions covering field data types, we refer readers to the README file in the shared Zenodo record [14]

#### REDVID detector geometry

**Fig. 1:**
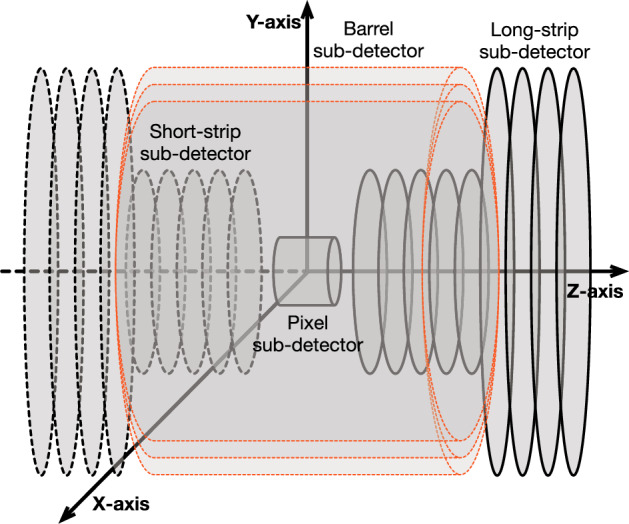
The fully parametric detector geometry, allowing for inclusion/exclusion of different sub-detector types, with full control over sub-layer counts, sizes and placements

At its core, a detector model is comprised of the geometric definitions of the included elements, shapes, sizes, and placements in space. Although we can support a variety of detector geometries, the overall structure, especially for our experimental results, resembles the ATLAS detector. Accordingly, there are four sub-detector types, *Pixel*, *Short-strip*, *Long-strip* and *Barrel*. The pixel and the barrel types have cylindrical shapes with the pixel being a filled cylinder, while the barrel being a cylinder shell with open caps. These are not hard requirements, as the geometry is fully parametric, and differing definitions can be opted for, e.g., a pixel as a cylinder shell. The long-strip and the short-strip types are primarily intended as flat disks, but can be defined as having a thickness, rendering them as cylinders. Sub-detector types can be selectively present or absent. Figure 1 depicts a representative variation of the detector geometry involving the aforementioned elements.

Structurally speaking, in a real-world detector, like ATLAS, the internals of short-strip and long-strip sub-detector types are different. We on the other hand, reduce such complexities to placement location and size, i.e., distance from the origin and sub-detector disk radius. Note that our geometric model does support disk thickness, which basically would turn disks into shallow cylinders. However, we have considered flat disks for our experiments.

#### REDVID event simulation

One of the simplifications for our complexity reduction approach is to consider a single collision per event (no pile-up), either aligning exactly to the origin point of the detector geometry, or at the presence of smearing for this alignment. This can be configured. However, the list of complexities, even without the polluting effects of multiple collisions, is extensive. Particles travelling through the detector matter could lead to secondary collisions, resulting in drastic changes in their trajectory. Such secondary collisions could also lead to the release of particles not originating from the collision event itself. These will show up as tracks with unusual starting points within the detector space, rather distant from the collision point. Some particles could also come to a halt, which would be seen as abruptly terminating tracks. Such physics-accurate behaviour of particles interacting with the present matter in detectors is not considered for our simulator. It must be noted that the generation of tracks originating far away from the origin and prematurely terminating tracks, can be emulated in our simulator in a randomised fashion.

### TrackML data set

To move forward in the complexity spectrum, beyond the “50–100 helical tracks REDVID” data set, we have opted to switch to the data associated with the TrackML Kaggle challenge [24]. Reduced versions of this data set are frequently used to assess the state-of-the-art models for this task. Released in 2018, the goal of this challenge was to identify machine learning solutions to the track reconstruction problem.

The TrackML data set is a simulated collection of proton-proton collision events. It simulates specifically the production of top quark–antiquark pairs in the high-pileup conditions expected at the HL-LHC, with each event incorporating an average of 200 pileup interactions. The simulated detector is designed as a generalised representation of an LHC tracker, featuring discrete layers of cylinder and disk-shaped sensor arrays operating within a magnetic field as layers. The data set provides 3D hit positions along with truth-level information about the particles responsible for generating these hits. Some simplifications with respect to more realistic data were made. For example, it does not account for reconstructed hit merging, as such occurrences are rare (less than 0.5%). Moreover, it excludes complex smaller detector elements such as electronic systems, cooling tubes, or cables.

The TrackML data set consists of 3D hit coordinates in a Cartesian coordinate system, with the global Z-axis defined along the beam direction [55]. The data set contains ten values associated with every particle: particle identifier *pid*, the vertex of origin ($v_x, x_y, x_z$), the initial momentum (in GeV/c) along each global axis ($p_x, p_y, p_z$), the particle charge *q* and the number of hits this particle generated *nhits*. We make use of only four of those as track defining parameters: $q, p_x, p_y, p_z$. However, the momenta are transformed into the spherical coordinate system according to

$$\begin{aligned} p&= \sqrt{p_x^2 + p_y^2 + p_z^2}  \texttt {,} \\ \theta&= \textrm{arccos}\left( \frac{p_z}{p}\right) \texttt {, and} \\ \phi&= \textrm{arctan2}\left( \frac{p_y}{p_x}\right) \texttt {.} \end{aligned}$$

As a preprocessing step, in addition to converting the track parameters to another coordinate system, we normalise the data.

Though resulting from a simulation, the TrackML data has considerable scale. It contains 8 850 individual events with an average of 10 780 particles, between 6 898 and 16 197, per event, which could be interpreted as the average track count. It includes particles represented with as many as 28 hit points. At the same time, there are particles with 0 associated hit points. The average particle hit count throughout the events is 8.52. The number of individual particle vertices composing a bunch crossing varies from 1300 to 3048. Substantial amount of *noise* hits are present (between 18 590 and 20 518 per event), which is one of the main factors making TrackML a challenging data set. Almost every publication considering this data set has performed some sort of noise reduction. For our highest complexity data set, “200–500 tracks per event from the TrackML data set”, we have selected between 200 to 500 tracks at random per event. This process is repeated five times, resulting in 43 725 reduced events. The tracks and hits are selected completely at random without any special attention to, e.g., number of noisy hits, i.e., we do not apply any noise reduction.

The TrackML data set is described extensively in literature. For further information please refer to [24] or [55].

## ML model designs

We cover four different designs, three based on the Transformer model architecture and one based on the U-Net model architecture. Note that the ease of evaluation is enabled by the reduction of complexity and simplified simulations.

### Transformer designs

The Transformer is a deep learning architecture that allows us to model pair-wise relationships among elements in sequential data by leveraging the attention mechanism [1]. It can be used to process sequences with permutation equivariance and work with variable input lengths, which makes it suitable for the task of trajectory reconstruction. Furthermore, due to the wide success of the Transformer architecture in various machine learning fields, many techniques have been invented and implemented that reduce its complexity to a sub-quadratic level, e.g., [57–61].

The model’s input sequence consists of *tokens* $\vec {x_1}, \vec {x_2},\ldots , \vec {x_n}$ and the initial embedding layer projects each token to a higher dimensional representation. Optionally, positional encoding is applied, to include the position of each token in the input into its representation. Next, the continuous values are fed into the encoder of the Transformer, which comprises a number of identical encoder layers. Each encoder layer consists of the multi-head attention operation, normalisation and a feed forward network, i.e., composed of fully connected layers.

The attention mechanism allows the model to embed the context of the entire sequence into the representation of each token. It operates using key (*K*), value (*V*), and query (*Q*) vectors, which are linear transformations of the input, derived from the input to the encoder block in the first layer and from the output of the previous layer in subsequent layers.

*Q* represents the elements for which we want to compute attention scores with respect to other elements in the sequence. The Key, *K*, provides information about the elements in the sequence, helping to determine their importance relative to the current element when computing attention scores. In self-attention, *K* represents the same elements as *Q*, but each is multiplied with different learned weights. The value, *V*, contains learned representations of the content of each element in the sequence.

In the scaled dot-product attention mechanism, the *Q* and *K* vectors are multiplied to compute attention scores, capturing dependencies within the sequence. These scores are then used to weight the *V* vectors, allowing the model to focus on the relevant information from the sequence. The attention function is given by,

$$\begin{aligned} \text {Attention}(Q, K, V ) = \text {softmax} \left( \frac{QK^T}{\sqrt{d_k}}\right) V\texttt {,} \end{aligned}$$

where $d_k$ is the dimensionality of the query and key vectors.

Typically, attention is computed on a set of queries at the same time, making *K*, *V* and *Q* matrices. Multi-Head Attention (MHA) allows for jointly attending to information from different representation sub-spaces at different positions in the data. MHA is summarised by

$$\begin{aligned} \text {MultiHead}(Q, K, V ) = \text {Concat}(\text {head}_1,\ldots , \text {head}_h)W\texttt {,} \end{aligned}$$

for which *h* is the number of heads, *W* is a parameter matrix, with $W \in {\mathbb {R}}^{hd_k\times d_{\text {model}}}$ and $d_{\text {model}}$ as the dimensionality of the embedding. The attention heads are used in parallel with dimensionality $d_k = \frac{d_{\text {model}}}{h}$ – this optimisation reduces the computational cost, making it comparable to that of single-head attention. Overall, the shape of the *K*, *V*, *Q* matrices is then $(B \times h \times N \times \frac{d_{\text {model}}}{h})$, equivalent to $(B \times h \times N \times d_k)$, with batch size *B*, number of attention heads *h*, and sequence length *N*.

The decoder has a similar architecture to the encoder, with the additional operation of multi-head attention over the output of the encoder stack. Its purpose is to generate sequences, and it is auto-regressive, meaning that it makes use of its previously generated symbols as additional input. The decoder takes a start token, its previous outputs and the encoder output as input, and generates the sequence’s next token as output. The multi-head attention layers employ masking to prevent it from conditioning on future tokens. Masking can also be utilised in the encoder, e.g., for ensuring the attention mechanism does not attend to padding values.

Note that the attention mechanism creates a $N\times N$ matrix for each attention head, for each encoder and decoder layer. This leads to a quadratic memory and time complexity of the Transformer and can restrict application for very long sequences, which has motivated research into optimisation techniques that reduce the cost of attention computations. Flash attention is one such method [62]: It splits *Q*, *K* and *V* into smaller blocks, loads them into fast static RAM, and only then computes the attention matrices with respect to these blocks. Each block’s output is scaled by an appropriate factor then added up, which leads to the same correct result as the normal attention mechanism ends up with. This approach boosts performance, with the authors reporting Flash attention as 3x faster and 20x more memory efficient than exact attention. Another technique that can optimise computational complexity involves using the Induced Set Attention Block from Set Transformers [63]. In that case, there is a set of inducing points, learnable parameters, which the high-dimensional input gets projected onto, which reduces the size of the attention matrix.

### U-Net design

The U-Net [2] is a CNN architecture that primarily segments images. It aims to classify each pixel in an image and allows it to distinguish and separate objects in complicated visual data. The “U” in U-Net represents its U-shaped structure: it has a contracting path (encoder) and a wide-ranging path (decoder) with a bottleneck in between. On the one hand, contextual information is caught by the contracting path through the progressive downsampling of the input image using convolutional layers and pooling operations that reduce the spatial dimensions and increase feature abstraction. This process allows the network to grasp the global structure of objects in the image while improving its analytical capabilities. The most abstracted features are extracted and the smallest resolution is reached at the bottleneck. On the other hand, the decoder part aims to reconstruct the image at the original resolution by employing transposed convolutions or upsampling layers. It gradually refines the output by combining contextual information from the encoder with finer details provided by the skip connections thereby ensuring that the spatial details lost during downsampling are satisfactorily recovered.

Although U-Net architectures are being widely used for image segmentation tasks it is important to note that traditional convolutional networks usually work on densely populated spaces. For most of the ordinary tasks this does not pose a real problem in terms of memory usage and computational effort. However, in problems where the nature of the data involves relatively sparse information spanning along very large physical spaces, modifications to traditional CNN may be considered. These adaptations can help mitigate physical memory limitations in hardware and accelerate the mathematical operations performed. More recently, a novel convolution operator named as Sparse Convolution (SC) has been introduced in order to work with sparse data which may represent a suitable option for processing event detection data in three-dimensional geometries, such as those proposed in this manuscript. Interested readers about how sparse convolutions work and how they are implemented are referred to [64] and the references therein.

### Design choices

**Fig. 2:**
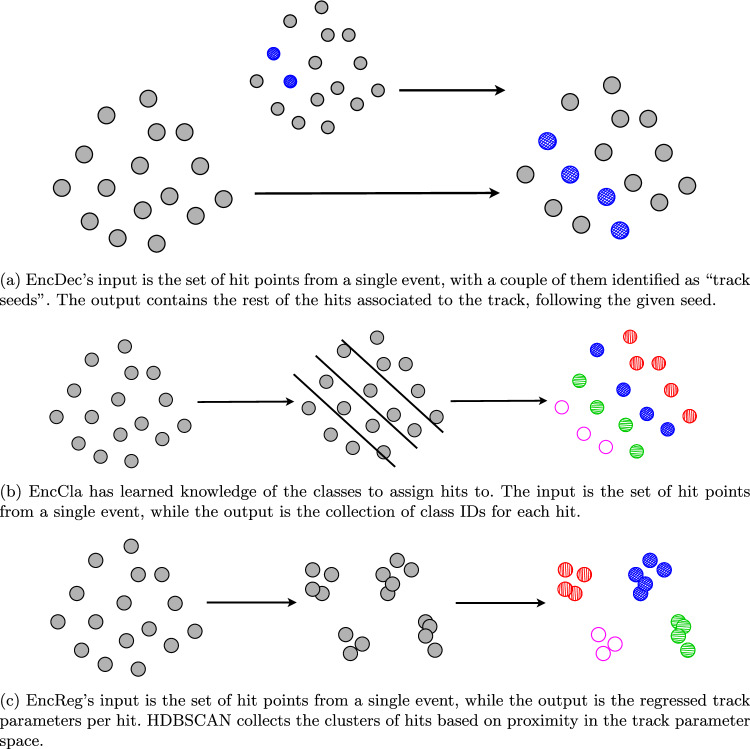
Intuitive visualisations of the inner-workings of three Transformer model designs, EncDec, EncCla and EncReg, respectively. We showcase in a simplistic manner the hit processing applied by each pipeline. Hits are represented by dots, with gray dots representing no track associations, and dots with the same colour belonging to the same track

The input sequence to the Transformers is a full event, with the tokens corresponding to hit coordinates (*x*, *y*, *z*), *which we do not discretise*. As a result of the unordered nature of the recorded hits, we do not use positional encoding for the Transformers. For models involving an *encoder-only* design, i.e., EncCla and EncReg, padding is used to allow variable length inputs. Note that different events could, for instance, have variable track counts, thus resulting in variable numbers of hits. The following sections will elaborate each model design and approach in detail. A simple depiction is presented in Fig. 2, covering Transformer-based model approaches, i.e., EncDec (Fig. 2a), EncCla (Fig. 2b) and EncReg (Fig. 2c).

#### Model 1 – EncDec

***Overall idea***

The overall idea of this method is similar to the approach used in autoregressive large language models, such as GPT [65], where the model predicts the next word in a sequence based on the preceding words. In our context, instead of predicting the next word, we predict the next hit from an initial set of hits. The model operates in an autoregressive manner, using previous predictions as inputs for subsequent predictions. This approach enables the model to sequentially build a complete track by iteratively predicting each hit based on the hits predicted so far. In contrast to GPT, we use an encoder-decoder design, which is well-suited for handling the sequential dependencies inherent in track reconstruction. This approach enables the model to sequentially build a complete track by iteratively predicting each hit based on the hits predicted so far.

***Details***

This model closely resembles the original Transformer architecture proposed by Vaswani et al. [1]. As such, it has an encoder and a decoder, which both make use of a self attention mechanism. The encoder encodes the full set of hits in a given event, and the decoder autoregressively predicts hits belonging to a particular track within the same event. Of particular interest are the differences with respect to the original Transformer architecture. Firstly, this model uses fixed-query attention [63] in the first encoder stack in order to ensure full positional invariance of the set of input hits. Furthermore, similarly to the encoder-only models in this paper, this model also omits positional encoding in the encoder, as the positions of hits are explicitly defined by the coordinates of the hits, which are fed to the model directly. The decoder does use positional encoding on the other hand, as for the constructed track the order of hits is relevant. The output format is also unique to this model, as rather than predicting a single token with SoftMaxed probabilities, the decoder outputs a length three vector with (*x*, *y*, *z*) coordinates of the next hit in the track.

***Advantages***

This model was tailored to the task of predicting the location of the next hit in a track given a set of prior input hits belonging to that track. As such, for this specific task, it achieves reasonable performance. It achieves 83% accuracy in predicting where a next hit is going to be within a 5% margin of error on the TrackML 10–50 tracks data set. With this in mind, it could potentially aid other tracking models, for example as a post-processing step to find missing hits for already constructed tracks, or to discard obviously wrong hits from already constructed tracks.

***Challenges***

This model differs from the others presented in this work due to the fact that it requires a seed (a short starting sequence of hits) from which to build the track. To do the full reconstruction from hits to tracks, it would thus require a preprocessing step to construct track seeds. Furthermore, whereas the other models in the present research reconstruct tracks in a one-step approach—at once creating all tracks in an event—this model builds tracks one by one. Building tracks one-by-one has the disadvantage that as soon as a single hit is predicted incorrectly, the model starts to diverge from the correct track path, and it is very unlikely to predict any following hits correctly. This leaves much more room for error, which is clear from the relatively worse FitAccuracy scores for this model in Table 1.

***Training notes***

This model was hyperparameter-optimised using the weights-and-biases platform [66]. The optimal model on the 10–50 tracks TrackML data set has 9 million trainable parameters.

#### Model 2 – EncCla

***Overall idea***

Here, we use the encoder component of the Transformer model as a classifier. It is important to note that the hits are not discretised at the beginning; instead, real values on a continuous 3D space are input into the model. Although discretisation may be beneficial in certain cases, our approach utilises the continuous nature of the data to improve model performance and accuracy. This encoder classifier design enables the model to effectively map hit data to corresponding track classifications, facilitating robust track reconstruction.

***Details***

This model takes a sequence of all hit coordinates from a single event as input, and outputs a sequence of class labels for each hit. The class label of a track is defined by discretizing the track parameter space into a fixed number of bins. This is done by binning each track parameter using a quantile-based approach, ensuring that each bin contains roughly equal number of hits. The class labels are created from all unique combinations of track parameter bins, each uniquely indexed.

The model has an input layer that projects hit coordinates into a higher-dimensional embedding space. It is followed by a number of encoder blocks and an output layer. To handle variable input lengths, the model uses padding up to the maximum number of hits in the current batch. Consequently, masking is used to ensure that the attention mechanism ignores the padding values. The output layer classifies each hit into a track candidate class by producing the probability distribution of all class labels for each hit and selecting the label with the highest probability as the predicted class. The hit is assigned to the track class with the highest probability, determined by the ArgMax of the SoftMax output of the classifier. The multi-class classification is trained using a cross-entropy loss function.

***Advantages***

The main advantage of the model is that it is a one-step model, assigning all hits to tracks in the full event in a single inference step. This makes its time complexity during inference lower than models that require a full inference step per hit prediction, such as the EncDec model.

***Challenges***

The model requires the a priori construction of track classes. This can only be done up to a finite granularity and can hinder clustering in high density environments.

***Training notes***

For the TrackML data set with 200–500 tracks, track parameters include *phi*, *theta*, *q*, and *p*. The model used 30 bins for *phi*, 30 bins for *theta*, and 3 bins for *p*. *q* values are binned into the only two possible values: − 1 and 1. All the combination of track parameter bins make up 5 400 track classes. The largest model has 1.5 million parameters.

#### Model 3 – EncReg

The third model under consideration is another encoder-only Transformer design. It is also a sequence-to-sequence model, the input of which is the hit coordinates of a single event. The output is the corresponding regressed track parameters. The model has an input layer identical to the previous model. The output is then of size (batches, max number of hits, number of track parameters). It is important to note that different data sets require different models, due to the difference in regressed parameters describing the tracks and data complexity due to increasing number of tracks.

To obtain the hit classification, a clustering algorithm is run on the regressed track parameter space. We make use of the HDBSCAN clustering algorithm, which can identify clusters of varying densities [67], and is relatively time efficient (in $O(n^2)$ [68]). It has two parameters, which we optimise for each specific data set.

Because of the large memory footprint of the attention mechanism and the constraints of the available GPU memory, the size of the events that we can work with is limited. We also identify that for larger events, training a single model is extremely costly in terms of time and computational resources. Therefore, for the largest data set utilised, we train two models: one with exact attention, and one with Flash attention (EncReg-FA), where measures are taken to improve the memory consumption of the attention computation, and speed up training and hyperparameter tuning. Moreover, for EncReg-FA, we also make use of mixed precision training, which the Flash Attention implementation relies on.^4^ The model’s weights, biases and gradients as well as the data passed to it are of type float-16 wherever possible, except during the operations which can greatly benefit from the data being of type float-32, i.e., values computed by large reductions such as batch normalisation. In addition to this being necessitated by Flash Attention, using lower precision in ML computation is another proven way of dealing with the bottleneck of GPU memory [69].

***Advantages***

Similarly to the EncCla model this is a one-step model, making its time complexity during inference lower than models that require a separate inference step per hit. Its speed makes it also suitable to be used as a refiner network, which regresses track parameters per cluster, i.e., per reconstructed track, and identifies falsely associated hits, increasing the purity of the cluster.

***Challenges***

Perhaps the biggest challenge for the EncReg model is the discovery of track parameters that sufficiently define a track and can be learned by the model. What coordinate system they should be in, dealing with angle symmetry, different weighting of the tracks’ contribution to the loss, etc., are some examples of things to consider. Another challenge is the evaluation of the model: as accuracy cannot be calculated for the regressed values, its performance is indirectly evaluated based on the formed clusters in the stage following it, or visual inspection of the regressed parameters plotted against the ground truth.

***Training notes***

The hyperparameters of the EncReg model are not fully optimised, but a brief search for suitable values is conducted. This Transformer has a learning rate of 0.001 and uses the Adam optimiser. Most models have 6 encoder layers, except the ones trained on the largest data used, TrackML 200–500 tracks, when there are 7 layers; each encoder layer has dropout of 0.1. The number of attention heads, embedding dimensionality and dimensionality of the fully connected layers are data set-specific. The largest model, EncReg-FA, has about 900,000 parameters.

#### Model 4 – U-Net

The last model implemented has supposed an alternative methodology to Transformer-like models. Due to the nature of the pixel regression task under consideration, the utilisation of a U-Net-based model appears to be a suitable option for a fruitful methodology. Vanilla U-Net models usually consider a set of the ordinary well-known dense convolutional layers, which are the core block building the network and where the major part of the computational effort occurs. For this use case, the input data should be preprocessed into a multi-dimensional tensor of size $(n_{\textrm{batches}},1,\textrm{width},\textrm{height},\textrm{depth})$, i.e., a three-dimensional tensor encompassing only one channel which contains discrete values of 0 and 1, indicating whether there is background or hit. Due to the sparse nature of this information, a more suitable U-Net model can be build if the convolutional operations are considered to occur directly on the sparse domain, thereby ignoring the background data governing the overall tensor representing the events. Consequently, since the hit occupancy for a certain event will be very low in general, the convolutional layers have been substituted by their sparse option [64, 70], thereby giving name to the sparse U-Net model that has been considered.

The task performed by this model is a classification process, being the original physical parameter space of the tracks binned according to a quantile-based procedure, using 30 quantiles, i.e., bins, per parameter. The output of the network is then the probabilities of belonging of each pixel to each class, i.e., to each bin. As a consequence, the vanilla Cross-entropy loss has been used as a cost function. Even though mixed precision can also be considered and implemented into this approach, there is not a real necessity since by focusing only on hit data using sparse tensors it is possible to save both memory and computational effort in convolutional operations. By this process, a label for each hit is obtained without the consideration of any post-clustering process.

***Advantages***

The model processes the entire event as a *sparse image*, meaning that it labels all hits (coordinates) at once without the need of attention layers or iterative pipelines. Despite their implementation is not straightforward due to the necessity of using sparse convolutions, attention procedures could be considered as a step forward leading to the construction of a modified U-Net architecture as seen previously in some literature [71]. The usefulness of sparse convolutional operations rely on both conceptual simplicity and computing speed. Studies such as [72] show state-of-the-art performance addressing particle two-dimensional space segmentation scenarios.

***Challenges***

Perhaps the most challenging aspect of this U-Net-based approach is the considered preprocessing. In the preprocessing step, when converting the original set of $\{x,y,z\}$ numerical values to the set of $\{i,j,k\}$ to create the sparse tensor of coordinates, a scale factor different from one can be considered. In addition, also during this step, it is possible to carry out an interpolation procedure to add more hits between the original ones. The scaling factor determines the physical spacing between the original points in the three-dimensional data. Conversely, the number of points used for interpolation between consecutive points enhances the amount of information provided to the network. Notably, these parameters were considered exclusively during the training process, leaving the test data unaltered. Different interpolation methods can be employed. In this case, the cubic interpolation method from the *SciPy library* has been utilised. As a consequence, both factors will introduce hyperparameters that need exploration, since a bigger scale factor will demand a higher amount of interpolated data, and vice-versa. However, a fixed scale factor of $\times 10$ and also 10 interpolated hits between each pair of original coordinates have led to the best results that are presented in this manuscript.

***Training notes***

Regarding the hyperparameters of the entire pipeline, one may expect the performance to vary with respect to different aspects. The first one refers to a certain arbitrary factor of scale that can be considered during the process of converting from the $\{x,y,z\}$ set to the $\{i,j,k\}$ one, while the second one would represent the considered amount of interpolated hits for each event. The largest model trained has a total of about 8 000 000 parameters. The said hyperparameters influence data processing but not the model itself. Since a balanced approach is desired, the training data was formatted as three-dimensional tensors for U-Net processing, with flexibility in scaling and interpolation. Various scaling factors ($\times 2$, $\times 5$, $\times 10$, $\times 20$) and interpolation levels (5, 10, 20, 50 points) were tested. A $\times 10$ scale with 10 interpolated points per hit provided the best trade-off between performance and training efficiency. However, it is important to note that these hyperparameters are not a priority, as they pertain to data preprocessing rather than the neural network itself.

### Model workflows

The model design elaborated above are functional within a workflow, hence the phrase “ML-assisted solution”. Depending on the model design, there may be simple or elaborate data pre/post-processing steps involved. In most cases, these pre/post-processing steps are the main factor differentiating computational performance for workflows. Detailed computational performance results are provided in Sect. 4.2.

**Fig. 3:**
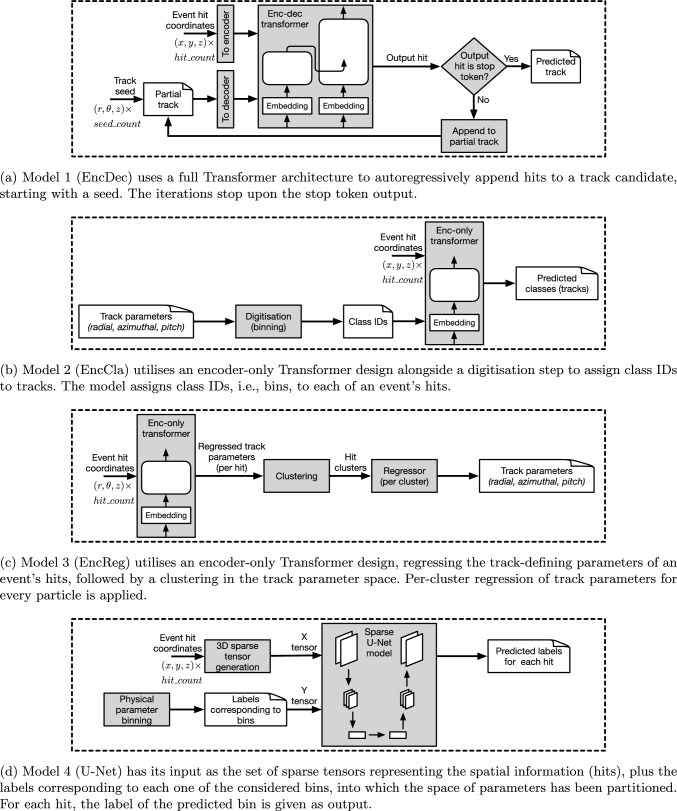
Depicting the high-level views of dedicated workflows for each ML model design

Accordingly, Fig. 3 depicts diagrams, covering steps and tasks within four individual workflows for our model designs, EncDec (Fig. 3a), EncCla (Fig. 3b), EncReg (Fig. 3c) and U-Net (Fig. 3d).

## Results and performance

### Prediction performance

To measure the *prediction accuracy* of our model designs, we consider a custom metric, named *FitAccuracy score*. It is essentially identical to the *TrackML score* [24], with a small modification that concerns the case of REDVID-generated data sets only. The TrackML score is calculated based on the association of weights to hits in the TrackML data. However, REDVID simulations do not generate particles and thus do not consider weights, but tracks. Essentially, we replace the true particle with the true track and consider the weight value of 1 for all hits to arrive at our custom FitAccuracy value. In this fashion, we can have a single comparable scoring for all data sets and model designs. In the definition of the TrackML score and, consequently, FitAccuracy, reconstructed tracks with four or more hits are considered, and at least 50% of a reconstructed track’s hits must originate from the same truth particle for that track to be considered for the scoring. The score of a track is the sum of correctly assigned hit weights. As such, the FitAcurracy score gives an indication of the fraction of correctly reconstructed tracks, and can thus be interpreted as a (weighted) reconstruction efficiency. Available scoring for each model is provided in Table 1.

**Table 1:** FitAccuracy scores for different models are given per data set, focusing on linear, helical and reduced TrackML data. Note that data sets can contain different fixed, or variable (randomised) counts of linear/helical tracks per event. Note that for the EncDec, the fitaccuracy score is slightly differently defined, due to the fact that it starts out with a seed. The seed hits are not counted towards the accuracy

Data set	FitAccuracy score				U-Net
	EncDec	EncCla	EncReg	EncReg-FA	
REDVID – 10–50 linear tracks	93%	93%	97%	–	68%
REDVID – 10–50 helical tracks	85%	93%	92%	–	62%
REDVID – 50–100 helical tracks	85%	88%	85%	–	57%
TrackML – 10–50 tracks	26%	94%	93%	–	–
TrackML – 200–500 tracks	–	78%	70%	67%	–

**Fig. 4:**
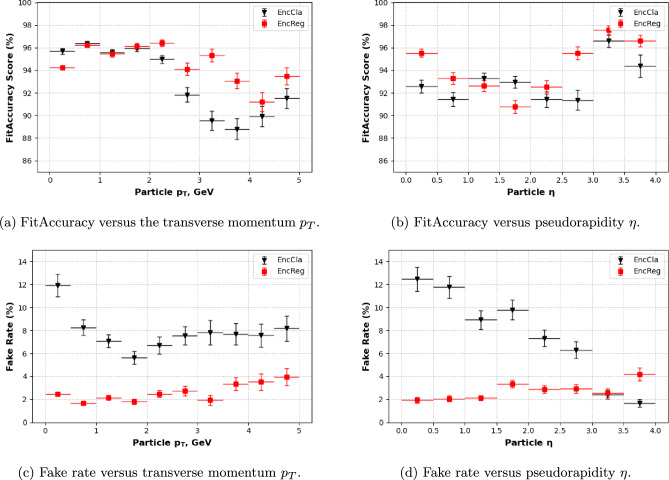
FitAccuracy score, as defined in Sect. 4, and the fake rate, which represents the fraction of predicted tracks that do not correspond to true tracks, as a function of physics observables for the EncCla and EncReg models on the TrackML 10–50 tracks data set, in black and red, respectively. (a) FitAccuracy as a function of the transverse momentum $p_T$, (b) FitAccuracy as a function of the pseudorapidity $\eta$, (c) fake rate as a function of the transverse momentum $p_T$, and (d) fake rate as a function of the pseudorapidity $\eta$. The uncertainties represent the 68% confidence intervals and were calculated via a bootstrapping procedure. The decreasing trend of EncCla in (d) is due to the increase of bin size with $\eta$, which affects both the number of correctly predicted tracks and the total number of predicted tracks

**Table 2:** Lists detailed computational effort consisting of CPU-time and GPU-time collections for every data set and model combination. For both measurements, mean instance inference time is provided. For the case of EncDec as an exception, we provide the wall-clock collection, i.e., the execution duration. This model is slower than the alternatives, and this is sufficiently visible with the execution duration

Data set	Model	Inference (mean) CPU side	Inference (mean) GPU side	Inference (mean) Wall-clock
REDVID 10–50 linear tracks	EncDec	n/a	n/a	41 s
	EncCla	0.1 ms	4.0 ms	–
	EncReg	8.3 ms	2.4 ms	–
	U-Net	8.5 ms	2.4 ms	–
REDVID 10–50 helical tracks	EncDec	n/a	n/a	19 s
	EncCla	0.1 ms	4.1 ms	–
	EncReg	8.7 ms	2.3 ms	–
	U-Net	8.6 ms	2.4 ms	–
REDVID 50–100 helical tracks	EncDec	n/a	n/a	27 s
	EncCla	0.1 ms	4.3 ms	–
	EncReg	18.6 ms	4.1 ms	–
	U-Net	20.4 ms	5.6 ms	–
TrackML 10–50 tracks	EncDec	n/a	n/a	16 s
	EncCla	0.1 ms	4.0 ms	–
	EncReg	5.8 ms	2.2 ms	–
	U-Net	n/a	n/a	–
TrackML 200–500 tracks	EncDec	n/a	n/a	–
	EncCla	0.1 ms	7.0 ms	–
	EncReg	70.5 ms	31.9 ms	–
	EncReg-FA	72.2 ms	3.6 ms	–
	U-Net	n/a	n/a	–

The models with the best overall performance, EncReg and EncCla, were further analysed to assess their physics performance as functions of two observables in the TrackML 10–50 tracks data set: pseudorapidity $\eta$^5^ and transverse momentum $p_T$. To evaluate model performance, two representative metrics were chosen: the FitAccuracy score, as defined in Sect. 4, and the fake rate, which represents the fraction of predicted tracks that do not correspond to true tracks. The results are presented in Fig. 4. Both models exhibit similar trends in their FitAccuracy scores, with EncReg showing marginally better performance at higher $p_T$ values. However, the fake rate analysis reveals a more pronounced difference: the encoder classifier (EncCla) performs significantly worse across most slices of phase space, except at high $\eta$.

### Computational performance

Beyond FitAccuracy, another important aspect to consider when comparing model designs and relevant workflows, is the computational effort required. The cost of inference is especially interesting, since tracking is bound to be deployed in an online (embedded in the data-taking pipeline), or semi-online fashion, and it is considered to be a time-critical use-case. Considering the workflows depicted in Fig. 3, we separate the computational effort for each model as the CPU side and the GPU side. Each data set, and model combination will have a different cost, as the processing and in most cases, the model training/inference, is dependent on the scale and complexity of data. Although following the same base design, often the model size has to match the size and the complexity of the considered data set.

Table 2 lists the complete set of metric collections for workflow blocks executed on CPU and for training/inference loops executed on GPU, in CPU-time and GPU-time, respectively. The GPU-time measures are given as mean iteration costs of the inference loop. Note that for the EncDec model, only wall-clock times were measured, as these were anyway significantly larger than for the other models. We omit the first iteration in training and inference loops to avoid cold-start effects invoking excess delays. All Transformer models have been trained on an NVIDIA A100 GPU with 40 GB HMB2 and 18 CPU cores, on the Snellius supercomputer.^6^ The training of the sparse U-Net model, using a similar hardware platform, has been done on an A100 GPU with 40 GB of memory from the Artemisa cluster.^7^

**Fig. 5:**
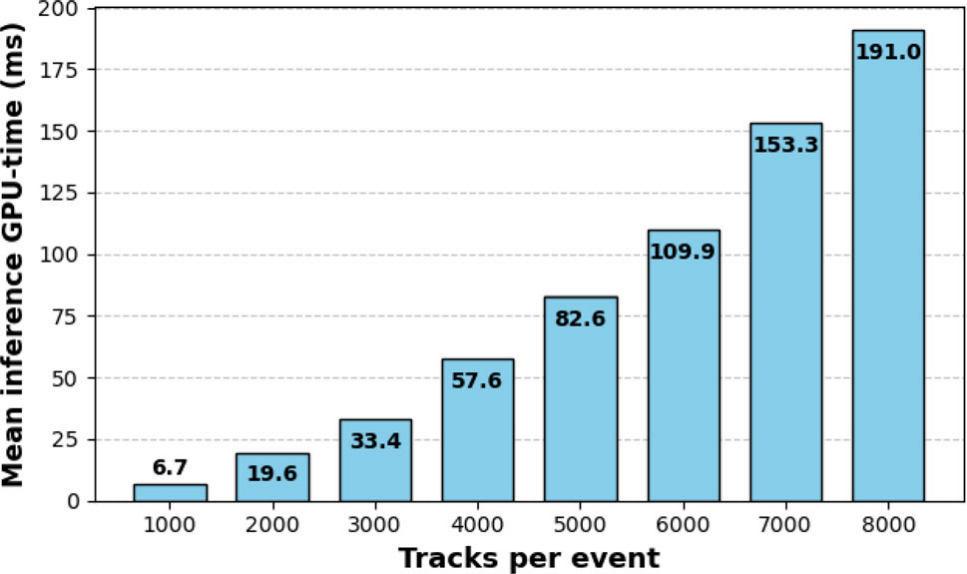
Mean inference GPU-time for the EncReg-FA model trained on the 200–500 tracks per event data set, plotted as a function of the number of tracks per event

Table 2 can be used to infer a rough estimate for the scaling of the mean inference GPU-time as a function of the input size. However, a more detailed analysis was conducted for the EncReg-FA model, trained on the 200–500 tracks data set. This model is particularly well-suited for scaling to larger events due to its integration of FlashAttention. The results, presented in Fig. 5, demonstrate that while the inference GPU-time does not scale linearly with input size, its growth remains sub-quadratic. Notably, even for events with 8000 tracks, the mean inference time remains below 200 ms, which is promising for applications to high-luminosity LHC data, where similar track multiplicities are anticipated. It is worth noting, however, that for events with higher track counts, larger models might offer improved physics performance, albeit at the cost of increased execution time. Consequently, this study should be viewed as a rough demonstration of scaling under the specific constraint of fixed model size. We do recognise that input scale is only one dimension of complexity and Fig. 5 intentionally does not take into account model prediction qualities, i.e., physics accuracy. The main point conveyed is that the indicated increase in *inference* computational burden is superior to traditional algorithms.

## Discussion – applying the method

**Fig. 6:**
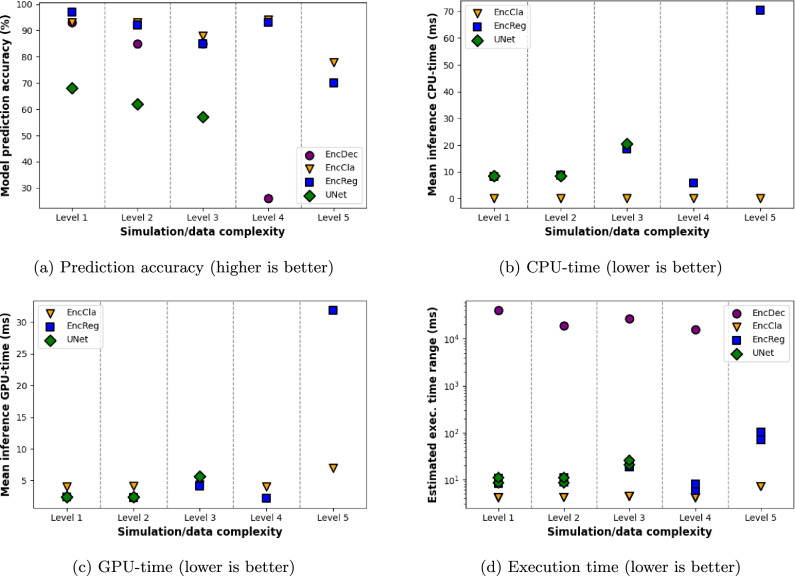
The five levels of simulation/data complexity in these plots, from simple to complex, correspond to the five data sets we have considered, in the same order as provided in Sect. 2. The combination of prediction accuracy and computational cost figures will be considered for early on model elimination. (a) Prediction accuracy results for four model designs at different complexity levels, (b) The CPU side of the computational effort for different model designs at different complexity levels, given as mean inference CPU-time, (c) The GPU side of the computational effort for different model designs at different complexity levels, given as mean inference GPU-time, (d) Providing an estimated range for overall inference execution time per event, based on collected CPU-time, GPU-time and wall-clock measurements. Note that an inference instance for EncDec will predict the next hit in a track, making a full event inference costly. Mean wall-clock measurement is provided for EncDec instead of a range based on CPU- and GPU-times

Next, we examine the performance of our different designs. Based on the two collected metric types, i.e., the model prediction accuracy results shown in Fig. 6a and the computational cost results for CPU and GPU, shown in Fig. 6b and c, respectively, we can look at how the models perform on different complex tasks. The total execution times provided in Fig. 6d are given as a range for EncCla, EncReg and U-Net models, while EncDec is represented by wall-clock time collection. This is due to implementation differences. We consider the maximum of CPU-time and GPU-time as the lower bound and the sum of the two (CPU-time and GPU-time) as the upper bound for this range, which is an acceptable estimation. Note that under parallel operation of a given computing platform, the CPU and the GPU activity is not necessarily sequential and the lengthiest of the two would be the least execution duration, hence the consideration of a lower bound.

***Encoder-Decoder (EncDec) model***

The first observation is that the Encoder-Decoder model takes the most time due to its autoregressive operation. This model does an inference step per hit, which significantly increases computational time with respect to the other models, which classify all event hits in a single inference step. Additionally, its performance declines with higher complexity tasks, specifically those involving more than 100 tracks or the reduced TrackML data. To mitigate excessive training times, this model was no longer used for the TrackML 200–500 tracks data.

***Encoder-Classifier (EncCla) model***

The EncCla model is the fastest solution, primarily operating on the GPU and producing track classes in a single inference step. The inference time is influenced by the size of the model, the attention matrices and the number of hits per event. Furthermore, the EncCla was found to be the best-performing model, delivering high accuracy without the need for any pre or post-processing steps. This model proved to be effective across all levels of task complexity.

***Encoder-Regressor (EncReg) model***

The EncReg model extends the Encoder-Classifier model by adding the time required to cluster hits into tracks, making it slightly more time-consuming. Additionally, the accuracy of the EncReg model appears to be slightly lower than that of the EncCla model. Despite these differences, the EncReg model is suitable for all simulation complexities.

***Sparse U-Net model***

The U-Net model can also determine the track classes for all hits in one step. The inference time increases slightly with larger input matrices and model sizes. However, it was not possible to achieve good accuracy for simulations with higher complexity levels (above level 3). This method was proposed as an alternative to Transformer-based models with attention mechanisms, but its performance was found to depend on parameters such as input image scale and the number of interpolation points in the training set. The REDVID data sets did not require sparse convolutional networks, but this approach ensured scalability for TrackML data. In addition, implementation restrictions of attention mechanisms within the *spconv* [73] package hindered direct comparisons with Transformers. This limitation restricts the U-Net model’s applicability to less complex scenarios.

By evaluating these results, we can better understand the trade-offs between computational cost and performance accuracy for each model, enabling us to choose the most suitable architecture for different particle tracking scenarios.

While negotiating the full scale and complexity of a realistic detector data set requires further research, our methodology provides an effective approach. To recapitulate, instead of picking one ML model design and iteratively improving it, we have opted for multiple designs. The layered approach to complexity allows for speedy evaluation and elimination of bad designs, making the design journey rather cost-effective. The way forward from this point on is to move forward in the complexity spectrum, represented by data sets of higher complexity and higher scale. In this particular case, models EncCla and EncReg, or improved variations of these shall be considered. Further fine-tuning along the way, in the form of post-processing models or computational steps could be considered. In short, we have the opportunity to try many design candidates, precisely because we can do it in a cost-effective way.

**Fig. 7:**
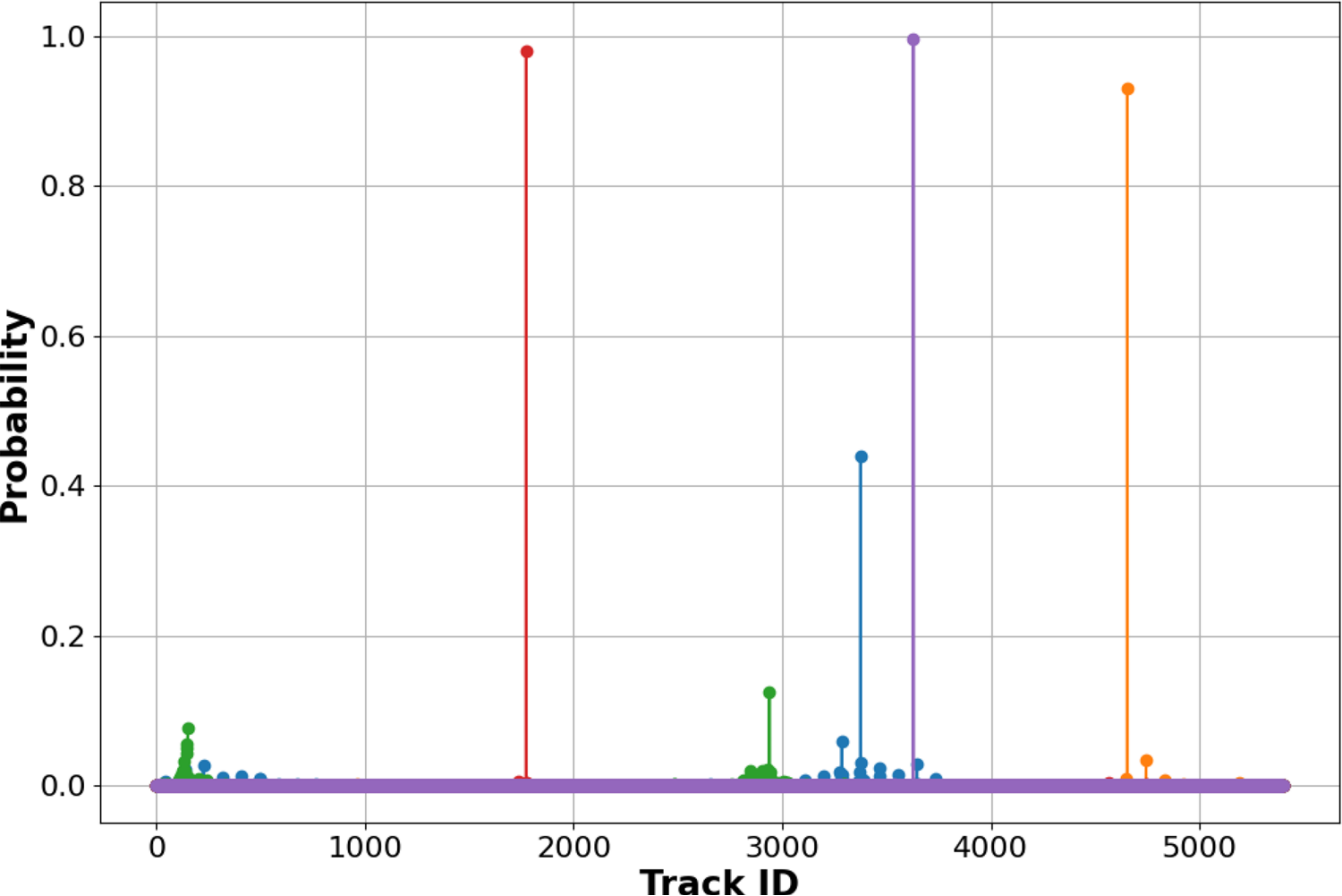
SoftMax outputs of the EncCla model for the first five hits for one event for all track classes, showing some hits with high confidence predictions for a track ID and others with low confidence predictions

## Conclusion and future work

***Model architectures***

Our main concluding remark relates to the effectiveness of the Transformer architecture for the task of particle tracking. Our experiments show potential for single-step processing of event hit data, especially with the encoder-classifier architecture. This architecture is inspired by a single-step translation of hits into track candidates. The low computational effort for the CPU side and the GPU side of event inference and good accuracy, shown in Fig. 6, are strong indications for this. The total inference time for the assignment of track classes to approximately, 10 000 hits per event (level 5) was estimated at 3–6 milliseconds (with or without the use of flash attention). It is not possible to directly compare our inference time with other approaches, as the data sets and computer architectures are not identical. However, we would like to point out again that the Kalman filter pipeline in [23] required 12 s of CPU-time, and an optimised Kalman filter algorithm required 1.8 s of CPU-time per event. A benchmark for GNNs is [34] and reports 2.2 s wall-clock time (including data transfer to GPUs, pre and post-processing steps) for the full TrackML events using an NVIDIA A100 GPU. This emphasises that the one-step transformer-encoder-classifier architecture is worth a closer look and needs to be further investigated to improve its performance through post-processing and preprocessing steps.

***Increasing complexity***

Another conclusion is the effectiveness of our method where we consider a complexity spectrum for the problem ranging from a simple to a complex reconstruction problem. Both the prediction accuracy and the computational effort results from Fig. 6 provide metrics for the early elimination of underperforming machine learning designs. It goes without saying that the design and validation effort (both human and machine) for lower complexity levels is incomparably lower than when tackling the problem in its full complexity. It should be noted also that the most complex data set considered in this work is still an order of magnitude removed from the true HL-LHC data in hit multiplicity. The scaling behaviour with increasingly complex data sets, as shown in Fig. 6 and Table 2, looks promising in particular for the EncCla model and to a lesser extent, for the EncReg model. However, a full evaluation of the included models up to the complexity of HL-LHC data is considered follow-up work, as significant adaptations would be required including the integration of pre- and post-processing steps into the algorithm, which may substantially optimise performance. Furthermore, computational developments, such as parallelised training and inference of the transformer models may also enable more efficient scaling.

***Future directions – post-processing models***

Our single-step models should be supplemented in future research by pre and post-processing steps. Pre-processing involves the formation of clusters from hits, i.e., one could envisage a combination of the encoder-regressor model to form clusters, which are then fed into the classifier model. There are many avenues for ML-based post-processing algorithms. Among these, one could again consider a combined application of the presented methods. For example, as the EncDec model is particularly well suited to the prediction of individual hits, it could be used as a post-processing step, where it could determine for each track candidate whether it is missing hits and add those hits to that track. Other additions could be models to detect incorrect hit assignments (per track candidate), to predict track parameters (with track candidate hit set as input) and to utilise physics-informed loss functions for an improved parameterisation. Note that our current solutions are limited to formation of hit clusters matching tracks and do not cover the actual parameterisation of a track function. It would scale with the number of track candidates (this would still be better than models that scale with the number of hit candidates) and would certainly increase accuracy.

***Future directions – faster models***

Other directions of future research could be oriented towards reducing the computational complexity of the Transformers through the use of recent developments in this field, for example using top-K attention, as suggested by Gupta et al. [74] and studying the large number of efficient transformer architectures, e.g., [57–61]. Regarding sparse U-Nets, while optimised for speed, future work could focus on further reducing computation time. This may involve studying the trade-off between performance and model size to find an optimal balance.

***Future directions – the use of the full posterior***

The EncCla provides also a unique feature that could be made use of in future developments, which is the fact that it does not just output for every hit exactly what track it belongs to, but it actually predicts a vector of probabilities per hits. As such, for each hit, it associates a probability to this hit belonging to all of the tracks. An example plot for five hits of an arbitrary event is given in Fig. 7. Such output will allow for powerful post-prediction analysis as an extra validatory step, resulting in corrections to the predicted hit associations if need be. In the context of language models, this full vector of probabilities has found extensive use, and similar strategies could be applied in this context.

## Data Availability

This manuscript has associated data in a data repository. [Author’s comment: The datasets generated during and/or analysed during the current study are available in the Zenodo repository, 10.5281/zenodo.14386134.]

## Footnotes

1 Also available at https://github.com/TrackFormers/TrackFormers-Models .

2 The CPU-time is multiplied by the HS06 factor of 17.8 for single-threaded execution, since the considered CPU is an Intel Xeon E5-2620v2. HS06 is a benchmark for measuring CPU performance in HEP. More information can be found at: https://w3.hepix.org/benchmarking/HS06.html.

3 For example, geometric constraints or a preprocessing algorithm such as Hough Transform [13].

4 This is a reliance imposed by the utilised framework, PyTorch.

5 Pseudorapidity is a widely used transformation of $\theta$, the angle relative to the beam axis, and is defined as $\eta \equiv -\ln \left[ \tan \left( \frac{\theta }{2} \right) \right]$.

6 https://www.surf.nl/en/services/snellius-the-national-supercomputer.

7 https://artemisa.ific.uv.es/web/.

## Code Availibility Statement

This manuscript has associated code/software in a data repository. [Author’s comment: The code/software generated during and/or analysed during the current study is available in the Zenodo repository, 10.5281/zenodo.14388535, and in the GitHub repository, https://github.com/TrackFormers/TrackFormers-Models.]
